# Widal's Reaction

**Published:** 1902-03-29

**Authors:** 


### WIDAL'S REACTION.

At the last meeting of the Clinical Society Dr. Hale White contributed a paper by himself and Mr. Pakes in which he described a case of malignant endocarditis which gave Widal's reaction. The blood on June 10th had failed to agglutinate typhoid bacilli even in a strength of 1 in 2; but on June 20th agglutination was produced with strengths down to 1 in 20, but not with one of 1 in 200. From the blood pure cultures of streptococcus longus were obtained, and on June 29th cultures from the urine showed the presence of a streptococcus identical with that previously obtained from the blood and a bacillus belonging to the colon group, but every attempt to isolate the typhoid bacillus failed. On September 13th the patient died from cerebral embolism. Blood taken from the heart and spleen showed streptococcus longus in pure culture, but no typhoid bacilli could be discovered. The patient never had spots or other signs of enteric fever, and on *post-mortem* examination no signs of healed ulcers were found. The authors pointed out the importance of this observation in regard to the diagnosis between typhoid fever and endocarditis, in the latter of which diseases many observers had failed to obtain Widal's reaction, for here was an instance of a patient who, although suffering from malignant endocarditis of streptococcal origin, without any clinical, *post-mortem*, or bacteriological evidence of typhoid fever, nevertheless developed in her blood-serum certain substances which had the power of agglutinating the typhoid bacillus and thus of simulating Widal's reaction for typhoid fever. In this case it was evident that the presence of the reaction could not be due to the patient having had typhoid fever previously, for no reaction was obtained when she was admitted. It should be added that in making the examinations in this case Widal's reaction occurred with four out of the five strains of bacilli which were employed. In replying after the discussion, Dr. Hale White pointed out that before a case in which agglutination occurred was allowed to invalidate the test it must be absolutely determined that the patient had never had typhoid, for the reaction had been found to take place when a patient had had typhoid as many as 23 years before.

